# Assessing the knowledge, attitudes, and psychological health of people who use drugs towards Hepatitis C: a cross-sectional study

**DOI:** 10.3389/fpubh.2025.1618440

**Published:** 2025-10-31

**Authors:** Yuanyuan Liu, Xiaoyan Zhang, Zhen Cao, Shanshan Zhu, Jianjun Fu

**Affiliations:** Department of Infectious Diseases, The Affiliated Xi'an Central Hospital of Xi'an Jiaotong University, Xi'an, Shaanxi Province, China

**Keywords:** chronic Hepatitis C, HCV knowledge, psychological distress, HCV awareness, risk behavior

## Abstract

**Introduction:**

Understanding the knowledge, risk behaviors, and psychological health of people who use drugs (PWUDs) is critical for Hepatitis C (HCV) elimination. This study aimed to assess these factors among PWUDs in a Drug Rehabilitation Center by comparing HCV-positive and HCV-negative individuals.

**Method:**

A total of 127 participants over 18 years (58 HCV-positive and 69 HCV-negative) were selected from the Lantian Drug Rehabilitation Center from April to June 2023. Data were collected through a descriptive, cross-sectional questionnaire (HCV awareness, HCV knowledge, SCL-90 tests) and laboratory blood tests, with which inferential and descriptive statistical analyses were performed.

**Results:**

Of the 127 participants, 58 (45.7%) were HCV-positive. A significant portion of both HCV-positive (31.0%) and HCV-negative (66.7%) individuals were unaware of their status prior to testing (*P* = 0.04). Overall HCV knowledge was high and similar between the two groups, though specific gaps persisted, with fewer than 40% recognizing the transmission risk from unregulated cosmetic procedures. Sharing syringes was significantly more prevalent in the HCV-positive group (*P* = 0.001). Compared to Chinese national norms, HCV-positive PWUDs exhibited significantly higher psychological distress across all ten SCL-90 dimensions (all *P* < 0.001), with the most pronounced elevations in somatization and psychoticism.

**Conclusion:**

Educational programs in rehabilitation centers appear effective in raising general HCV awareness, significant gaps in knowledge about community-based transmission routes persist. The profound psychological distress observed among HCV-positive PWUDs, coupled with the high rate of status unawareness, underscores the urgent need for integrated care models that address both virological treatment and mental health support.

## 1 Introduction

The hepatitis C virus (HCV), transmitted via blood exposure, is a major cause of cirrhosis and hepatocellular carcinoma (1). In 2015, the World Health Organization (WHO) estimated that approximately 71 million people worldwide were living with chronic HCV (2), with people who use drugs (PWUDs) accounting for more than 50% of infections (3). In China, the pooled prevalence of HCV among PWUDs was 71.6% (95% CI: 65.7%−77.6%) (4).

Direct-acting antivirals (DAAs) have revolutionized HCV management by providing high cure rates across all stages of infection, however, substantial deficiencies remain in the care cascade, as merely 21% are diagnosed and 13% treated (5). To meet WHO's elimination targets by 2030−90% diagnosed, 80% treated, and a 90% reduction in new infections—greater attention to high-risk populations is essential (5). Therefore, given that no effective vaccine is currently available, understanding the knowledge, attitudes, risk behaviors, and psychological health of PWUDs is fundamental to developing evidence-based prevention and control strategies (6). In China, among methadone maintenance treatment (MMT) patients, HCV seroprevalence was high (70%), yet knowledge was limited and treatment uptake low (13.7%), hindered by financial, educational, and policy barriers (7). Lower levels of HCV knowledge have been linked to diminished treatment willingness and decreased uptake of HCV-related health services in PWUDs (8, 9), while higher knowledge correlates with greater engagement and willingness to commence therapy (10, 11). Moreover, PWUDs often present with depressive symptoms (12), and HCV infection may further exacerbate psychological distress (8, 9). Therefore, expanding access to integrated HCV prevention, treatment, and mental health services is essential for meeting the WHO's 2030 elimination objective.

In this study, we aimed to explore the knowledge, attitudes, risk behaviors and psychological health regarding HCV among PWUDs in a rehabilitation center, comparing HCV-positive and HCV-negative individuals to assess differences in knowledge gaps and psychological burden. These real-world findings have direct implications for developing targeted interventions consistent with the WHO elimination strategy.

## 2 Methods

### 2.1 Recruitment and ethical approval

Participants were recruited between April and June 2023 in a drug rehabilitation center in Lantian District, Shaanxi Province, China. In line with the Anti-Drug Laws enacted in China, Chinese residents, aged between 18 and 65 years, who were newly identified as illicit drug users and diagnosed with illegal drug abuse or dependence according to DSM/ICD criteria, were admitted to a medical facility for an evaluation of their addiction severity (13). Following this assessment, they underwent detoxification treatments, which were administered under the guidance of community social workers outside the hospital setting. The rehabilitation center regularly provides PWUDs with education to enhance knowledge of HCV prevention and management. Exclusion criteria were: a current diagnosis of an acute psychosis. The study was conducted following the Declaration of Helsinki, and all PWUDs who agreed to participate to the study provided written informed consent prior to data collection. Participants provided consent by completing and submitting the survey, which required approximately 20–30 min. Upon completion, each participant received free HCV antibody and HCV-RNA testing (total value: ¥460) as reimbursement for their participation. The study was approved by the Medical Ethics Committee of Xi'an Central Hospital (No. LW-2024-017).

### 2.2 Data collection instruments

This study adopted a cross-sectional design with targeted recruitment of PWUDs. Data were collected using a structured questionnaire developed by a steering committee of hepatology and psychiatry experts, along with the validated Symptom Checklist-90 (SCL-90, Chinese version, 1984). Participants were asked to report their awareness of any prior HCV diagnosis on the questionnaire. After completing the questionnaire, all participants received free serological HCV antibody and HCV-RNA testing for confirmation and definitive diagnosis. Laboratory-confirmed HCV status was used to divide participants into positive and negative groups for comparison. Our study was designed to capture knowledge, attitudes, and psychological health in a mixed post-diagnosis and status-unaware setting among PWUDs, which reflects the real-world conditions in which educational and support interventions typically occur.

### 2.3 Demographic characteristics

The following items were collected: sociodemographic characteristics, including name, age, gender, education level (Low and Primary school, Junior high school, Senior high school, University and high above), residence (rural, urban), alcohol history, smoking history, awareness of HCV status, as well as HCV RNA and HCV genotype. None of the HCV-positive participants were receiving DAA therapy.

### 2.4 Knowledge of HCV prevention and treatment

The questionnaire was designed and finalised by a steering committee comprising of hepatology experts and psychiatrists from the Xi'an Central Hospital. We assured participants that the study was voluntary, anonymous, and confidential, including a statement that the purpose of the study was to assess participants' knowledge, risk behaviors, and psychological symptoms related to the topic of investigation.

To assess participants' understanding of HCV prevention and treatment, we designed a structured knowledge questionnaire section comprising eight core items. These questions were adapted from established national guidelines and validated HCV knowledge surveys, and were reviewed by hepatology experts for content relevance. Items assessed awareness of common and less common HCV transmission routes (e.g., sharing syringes, blood transfusions, casual contact, tattooing/piercing, and sexual behaviors), Each response was coded as correct or incorrect based on WHO and Chinese CDC recommendations, where non-recognition of sexual transmission risk was considered incorrect despite this being a less frequent route of exposure. The potential for chronic HCV to progress to cirrhosis or hepatocellular carcinoma, and perceptions of HCV curability. Each question was presented in a dichotomous format (“Yes/No” or “Agree/Disagree”), and responses were coded as correct or incorrect according to current WHO and Chinese Center for Disease Control and Prevention recommendations. The knowledge assessment was administered in face-to-face sessions by trained healthcare workers in the rehabilitation centre to ensure comprehension and completeness.

### 2.5 Behavioral characteristics of HCV

Behavioral characteristics related to potential HCV risk exposure were evaluated using a structured set of eleven items. These items were designed to capture both self-reported clinical symptoms and engagement in behaviors that may increase HCV transmission risk. Participants were first asked about the presence of common HCV-related clinical symptoms, including fatigue, gastrointestinal discomfort (e.g., nausea, abdominal pain, bloating), jaundice of the skin or sclera, and other relevant symptoms. Subsequent items assessed whether participants had undergone invasive procedures in informal or unregulated settings (e.g., tattooing, piercing, liposuction, pedicures), received dental procedures in private clinics, undergone endoscopic examinations, or received blood transfusions or blood products. Participants were also queried about high-risk practices such as syringe sharing and engagement in commercial sexual activities. Responses were recorded in a dichotomous format (“Yes/No”), and each risk behavior was treated as a categorical variable. Data collection was conducted in a face-to-face interview format by trained research staff to ensure comprehension and accurate reporting. To reduce potential social desirability bias, confidentiality was emphasized prior to questionnaire administration. The internal consistency of the HCV Knowledge Questionnaire was examined using the Kuder–Richardson Formula 20 (KR-20), yielding a coefficient of 0.89, which indicates excellent reliability. Construct validity was further supported by a Kaiser–Meyer–Olkin (KMO) value of 0.84 and a significant Bartlett's test of sphericity (Bartlett's Test of Sphericity, *P* < 0.001). These indices demonstrate that the instrument possesses strong psychometric properties for evaluating HCV-related knowledge in this population.

### 2.6 Symptom Checklist-90 (SCL-90)

Psychological health was evaluated using SCL-90. This instrument delineates ten principal symptom dimensions, namely somatization (SOM), anxiety (ANX), obsessive-compulsive disorder (OCD), depression (DEP), interpersonal sensitivity (IS), psychoticism (PSY), paranoid ideation (PAR), hostility (HOS), phobic anxiety (PHOB), and the general severity index (GSI). For the SCL-90 (Chinese 1984 revision), the internal consistency reliability of the SCL-90 was assessed with Cronbach's alpha, which was 0.98 in this sample, demonstrating excellent reliability. Construct validity was supported by a KMO value of 0.823 and a significant Bartlett's test of sphericity (*P* < 0.01), and the results supported a nine-factor structure that aligns with the established theoretical dimensions of the instrument. These findings confirm that the SCL-90 demonstrated strong reliability and construct validity within the current sample.

### 2.7 Statistical analysis

We hypothesized that awareness and mental health differences existed between the HCV and non-HCV groups. All the data were collected and transferred to SPSS version 25.0 (IBM SPSS Statistics for Windows, IBM, Armonk, NY, USA, 2017) for statistical analysis. Descriptive statistics were used to summarize all demographic and awareness variables, presented as means ± standard deviation for continuous variables and frequencies (percentages) for categorical variables. Group comparisons between HCV-positive and HCV-negative PWUDs were conducted using χ^2^ tests or Fisher's exact tests for categorical variables, and Welch's *t*-test for continuous variables. Statistical analyses were performed with SPSS version 25.0 (IBM, Armonk, NY, USA). Statistical significance was set at *P* < 0.05 (two-tailed).

## 3 Results

### 3.1 Demographic characteristics

A total of 127 PWUDs participated in the study. Their demographic data are presented in Table 1. A majority of participants (60.63%) were aged 45 to 59 years, with the cohort ranging from 18 to over 65 years. High rates of alcohol consumption (93.7%) and smoking (95.28%) were prevalent across the sample.

**Table 1:** Demographics of the study participants.

**Characteristics**	**Total (127)**	**HCV (58)**	**Non-HCV (69)**	**Statistical difference (Fisher's exact test)**	***P***
	***N*** **(%)**	***N*** **(%)**	***N*** **(%)**		
**Sex**				Yes	0.00
Female	26 (20.47%)	13 (22.41%)	13 (18.84%)		
Male	101 (79.53%)	45 (77.59%)	56 (81.16%)		
**Age (years)**				No	0.67
18–29	6 (4.72%)	1 (1.72%)	5 (7.25%)		
30–44	31 (24.41%)	12 (20.69%)	19 (27.54%)		
45–59	77 (60.63%)	38 (65.52%)	39 (56.52%)		
>60	13 (10.24%)	7 (12.07%)	6 (8.70%)		
**Education**				No	0.30
Low and Primary school	2 (1.57%)	2 (3.45%)	0 (0.00%)		
Junior high school	39 (30.71%)	20 (34.48%)	19 (27.54%)		
Senior high school	60 (47.24%)	28 (48.28%)	32 (46.38%)		
University and high above	26 (20.47%)	8 (13.79%)	18 (26.09%)		
**Location**				No	0.63
Rural	102 (80.31%)	46 (79.31%)	56 (81.16%)		
City	25 (19.69%)	12 (20.69%)	13 (18.84%)		
**Alcohol**				No	1
No	8 (6.30%)	8 (13.79%)	0 (0.00%)		
Yes	119 (93.70%)	50 (86.21%)	69 (100.00%)		
**Smoking**				No	1
No	6 (4.72%)	3 (5.17%)	3 (4.35%)		
Yes	121 (95.28%)	55 (94.83%)	66 (95.65%)		
**Awareness of HCV status**				Yes	0.04
No	64 (50.39%)	18 (31.03%)	46 (66.67%)		
Yes	63 (49.61%)	40 (68.97%)	23 (33.33%)		

Based on their HCV status, participants were categorized as HCV-positive (*n* = 58, 45.67%) or HCV-negative (*n* = 69, 54.37%). For the HCV-positive cohort, the mean HCV RNA viral load was 1.34 × 107 IU/ml. Genotype 3a was most prevalent (60.34%, *n* = 35), followed by 3b (13.79%), 6a (10.34%), and 2a (8.62%). Genotype 1b (1.72%) and mixed 1b+3a (1.72%) were also found; two participants (3.45%) had an undetectable genotype. None of the participants in this group were currently receiving or had previously received DAA therapy. The two groups did not differ significantly in terms of age (*P* = 0.67), education (*P* = 0.30), location (*P* = 0.63), alcohol consumption (*P* = 1.00), and smoking (*P* = 1.00). However, a significant difference was found in the distribution of sex between the HCV-infected and non-HCV-infected PWUDs (*P* = 0.00). There was also a significant difference in participants' self-reported awareness of their HCV status (*P* = 0.04). A substantial portion of the HCV-positive group (31.03%) was unaware of their infection, while an even larger proportion of the HCV-negative group (66.67%) was unaware of their status.

### 3.2 General knowledge and awareness of Hepatitis C

**Figure 1:**
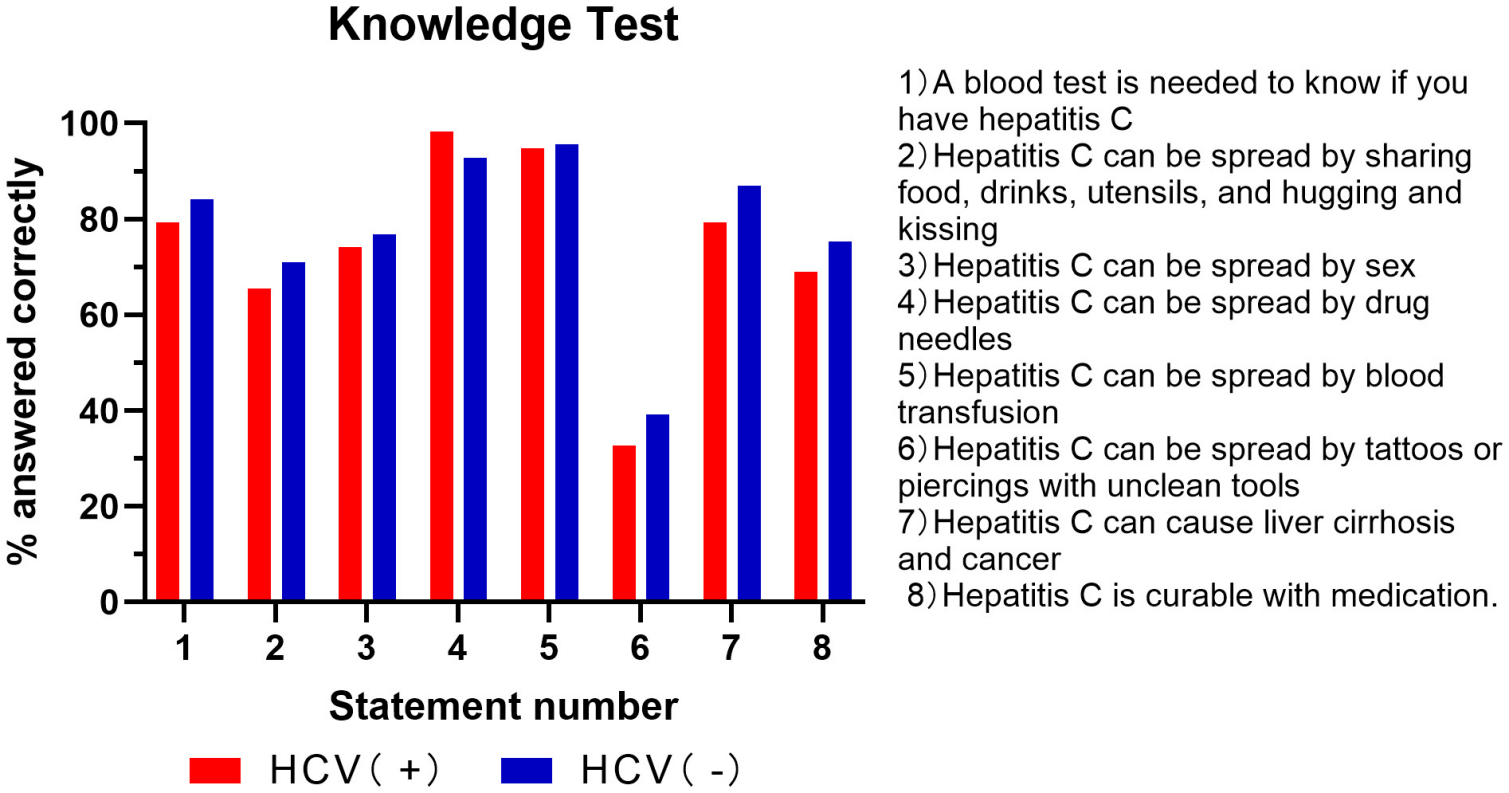
Percentage of correct answers on the Hepatitis C knowledge test in the HCV-positive and HCV-negative groups.

We assessed whether there were differences in HCV-related knowledge between HCV-positive and HCV-negative PWUDs. Non-HCV-infected PWUDs showed slightly higher awareness of the potential for HCV to remain asymptomatic (84.06%, 58/69) compared to those infected with HCV (79.31%, 46/58) (Figure 1, Item 1). The majority (86.96%, 60/69) were aware that HCV could increase the risks of developing liver cirrhosis and liver cancer, compared to those infected with HCV (81.03%,47/58) (Figure 1, Item 7). Awareness that hepatitis C is curable was high among both HCV-negative participants (75.36%) and HCV-positive participants (67.24%) (Figure 1, Item 8). However, there were no statistically significant differences in knowledge levels found between the two groups (all *P* > 0.05). This finding strongly suggests that the hepatitis C education programs frequently conducted at the drug rehabilitation center are effective and beneficial for both HCV-positive and HCV-negative PWUDs.

Regarding knowledge of HCV transmission routes, no statistically significant differences were observed between the HCV-positive and HCV-negative groups (all *P* > 0.05). Both cohorts demonstrated a high awareness of well-established transmission methods, including sharing injection equipment (HCV-positive: 98.28%; HCV-negative: 92.75%) and receiving blood transfusions from an infected person (HCV-positive: 93.10%; HCV-negative: 95.65%) (Figure 1, Item 4, 5). Awareness was also comparable for sexual contact involving blood exposure (HCV-positive: 74.14%; HCV-negative: 76.81%) (Figure 1, Item 3). However, a critical finding was the low awareness concerning transmission risks from tattoos, eyebrow tattooing, or ear piercings performed in street shops or small salons; only 32.76% of HCV-positive and 39.13% of HCV-negative participants recognized this risk (Figure 1, Item 6). This indicates that while educational programs at the rehabilitation center are effective for common transmission pathways among all PWUDs, regardless of HCV status, there is a clear need to enhance the dissemination of knowledge regarding the risks posed by unregulated cosmetic procedures.

### 3.3 Behavioral characteristic of Hepatitis C

**Figure 2:**
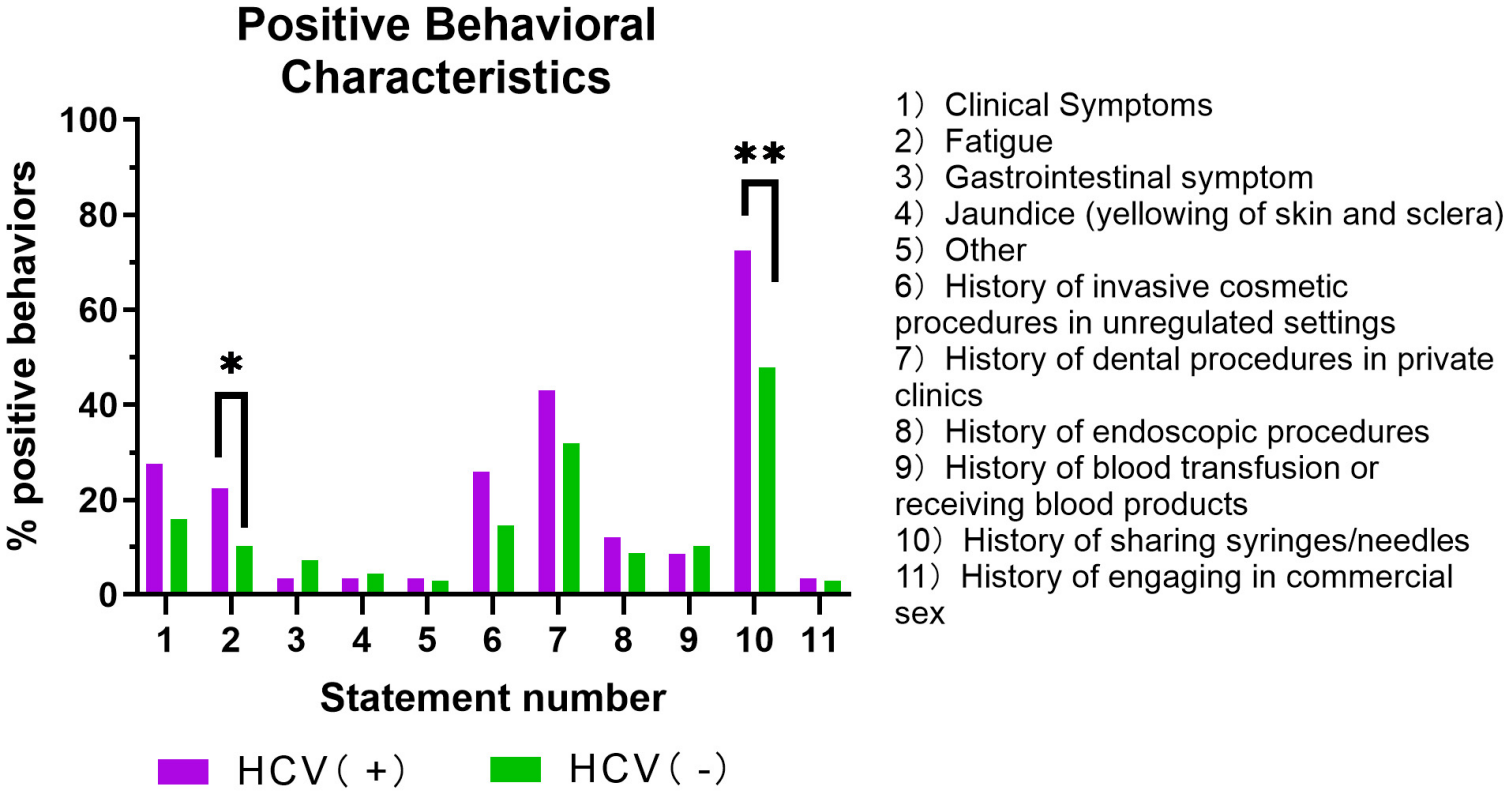
Percentage of positive behavioral characteristics in the HCV-positive and HCV-negative groups.

Regarding clinical presentation, fatigue was the most prominent symptom reported and was significantly more prevalent among the HCV-positive group than the HCV-negative group (*P* = 0.04) (Figure 2, Item 2). In contrast, other symptoms such as gastrointestinal issues and jaundice were reported by a small minority in both cohorts, with no statistically significant differences observed between the groups (Figure 2, Item 3, 4, 5).

In the analysis of potential risk exposures, the most critical differentiating factor was injection-related behavior. A history of sharing syringes or needles was significantly more common among HCV-positive PWUDs than among their HCV-negative counterparts (*P* = 0.001) (Figure 2, Item 10). Although a higher percentage of HCV-positive individuals also reported histories of invasive cosmetic procedures, dental procedures, and endoscopic procedure compared to the HCV-negative group, these differences were not statistically significant (Figure 2, Item 6, 7, 8).

### 3.4 The symptom checklist-90 assesses the mental health of PWUDs with HCV

This study used the SCL-90 to assess the mental health status of participants. As shown in the Table 2, HCV-positive PWUDs group (*n* = 58) exhibited significantly higher levels of psychological symptoms across all dimensions of the SCL-90 compared to Chinese national norms (*n* = 12,160) (all *P* < 0.001). Among these, somatization (2.27 ± 0.53 vs. 1.37 ± 0.46) and psychoticism (2.45 ± 0.57 vs. 1.34 ± 0.44) exhibited the greatest elevations, suggesting both physical distress and distorted thought processes were particularly salient. DEP, ANX, and IS were also prominently elevated. These results confirm that HCV-positive PWUDs experience significantly greater psychological distress than expected in the general population.

**Table 2:** SCL-90 scores (mean ± SD).

**Dimension**	**Norm (*n* = 12,160)**	**HCV (*n =* 58)**	***t***	***P***
SOM	1.37 ± 0.46	2.27 ± 0.53	−12.91	< 0.00001
ANX	1.51 ± 0.55	2.52 ± 0.61	−12.59	< 0.00001
OC	1.66 ± 0.58	2.44 ± 0.65	−9.12	< 0.00001
DEP	1.45 ± 0.53	2.36 ± 0.60	−11.53	< 0.00001
IS	1.51 ± 0.55	2.38 ± 0.59	−11.21	< 0.00001
PSY	1.34 ± 0.44	2.45 ± 0.57	−14.81	< 0.00001
PAR	1.41 ± 0.50	2.27 ± 0.74	−8.84	< 0.00001
HOS	1.48 ± 0.57	2.23 ± 0.67	−8.51	< 0. 00001
PHOB	1.23 ± 0.39	2.28 ± 0.65	−12.29	< 0.00001
GSI	1.51 ± 0.58	2.36 ± 0.58	11.13	< 0.00001

## 4 Discussion

In this study, unawareness of HCV status before laboratory testing was significantly higher among HCV-negative participants (66.67%) compared to their HCV-positive counterparts (31.03%) (*P* = 0.04). This difference is likely because the majority of HCV-positive participants had previously received a diagnosis through prior care, while the high unawareness among the uninfected group highlights a critical system-level gap: many high-risk PWUDs have not received clear, definitive test results, despite engaging in risk behavior. This suggests that even uninfected individuals may not fully understand the asymptomatic nature of HCV or the necessity of regular testing. Therefore, the need for expanded education and screening is urgent, especially since previous studies show that patients with HCV had significantly lower awareness rates if they were unaware of their infection route, lack HCV education (β = −1.648, 95% CI −2.224 to −1.073, *P* < 0.001), or were unaware of their diagnosis (β = −1.097, 95% CI −2.094 to −0.010, *P* = 0.031) (14). Furthermore, HCV-positive patients in China have been shown to have a poor understanding of HCV and negative attitudes towards other HCV-positive patients (12, 14).

Our study identified a high level of HCV knowledge among PWUDs in the rehabilitation center, which is particularly noteworthy when compared to the 47.6% awareness rate observed in the general population in China in 2023 (15). A key finding was the absence of a statistically significant differences in overall knowledge between HCV-positive and HCV-negative participants, suggesting that the educational efforts are reaching and benefiting both groups effectively in the Drug Rehabilitation Center. This is an encouraging outcome, as higher baseline HCV knowledge correlates with greater healthcare engagement and willingness to begin treatment (16), both of which are crucial for achieving HCV elimination goals. A noteworthy and seemingly paradoxical finding was that HCV-negative PWUDs occasionally demonstrated slightly higher awareness on specific points, such as curability of HCV(75.36% vs. 67.24%), and its potential to be asymptomatic (84.06% vs. 79.31%). This suggests that a diagnosis of HCV does not automatically translate into a more comprehensive understanding of the disease (12), indicating a disconnect between diagnosis and effective knowledge formation. This underscores the necessity for ongoing, tailored education even after a positive diagnosis to address specific misconceptions and reinforce key health message.

HCV is primarily a bloodborne pathogen spread chiefly through the reuse or improper sterilization of medical tools like syringes, unscreened blood transfusions, and shared injection equipment among PWUDs (15, 17). While it can also be transmitted from mother to child and through sexual activities involving blood exposure, these routes are less frequent (18, 19). Our study revealed that the educational programs appear highly successful in conveying the most critical risks associated with drug use. Both cohorts demonstrated high awareness of well-established transmission methods like sharing syringes and blood transfusion as transmission vectors. However, misconceptions about less common transmission methods, like sexual behaviors and getting tattoos or piercings in unregulated settings, persist. One study published in Hepatology indicates that there is no increased risk of sexual transmission of HCV among heterosexual couples in regular relationships. This risk increases among persons with multiple sexual partners (adjusted odds ratio [aOR] 2.2–2.9) (20). Fewer than 40% of participants in either group recognizing this risk. This finding is significant because while these routes may be less frequent than injection-related transmission, they represent a preventable source of new infections that is currently being overlooked in educational curricula. This indicates an urgent need to update and expand educational content to cover these common community-based risks, ensuring that prevention strategies are comprehensive and address the full spectrum of potential HCV exposure scenarios.

The analysis of behavioral characteristics provides a clear and compelling confirmation of the primary transmission driver of HCV within this cohort. The finding that a history of sharing syringes or needles was significantly more common among HCV-positive PWUDs than their HCV-negative counterparts (*P* = 0.001) aligns with extensive global evidence identifying injection drug use as the principal route for HCV transmission (21, 22). This result powerfully underscores the critical and ongoing importance of harm reduction strategies, such as needle and syringe programs, as the cornerstone of HCV prevention efforts in this key population. Although a higher percentage of HCV-positive individuals reported histories of invasive cosmetic, dental, and endoscopic procedures, these differences did not reach statistical significance in our sample. While this suggests that syringe sharing is the overwhelmingly dominant risk factor differentiating the two groups, the consistent trend across these other potential exposures indicates they should not be entirely dismissed. This is particularly relevant when considered alongside our other finding of low community awareness regarding risks from unregulated cosmetic procedures. These secondary routes may still contribute to HCV transmission, and they remain an important target for comprehensive public health education.

Regarding clinical presentation, the finding that fatigue was the only symptom significantly more prevalent in the HCV-positive group is clinically instructive. It highlights the often subtle and insidious nature of chronic HCV infection, which frequently lacks overt, specific symptoms like jaundice or gastrointestinal distress in its earlier stages. Fatigue is a well-documented and challenging symptom for patients with chronic HCV, often impacting quality of life even before severe liver disease develops. This reinforces the vital public health message that screening for HCV must be driven by risk-factor assessment rather than waiting for the manifestation of visible symptoms.

Historically, mood disorders, especially depression and fatigue are the most challenging symptoms with which HCV-related patients have to cope (23). HCV-related depression can be partially attributed to social and occupational limitations consequent on the diagnosis, including perceived restrictions in sharing food and sexual contact, great life stressors, and few social supports, especially intravenous drug use (24, 25). Comparably, PWUDs often endure excessive stress during compulsory detoxification treatment, which is currently the major drug rehabilitation modality in China, leading to a high prevalence of anxiety symptoms (26). In a study conducted in China, researchers used the SCL-90 scale (Chinese version) to assess the psychological health of PWUDs and compared it to that of healthy adults; the study found that PWUDs exhibited more severe psychopathological conditions across all ten dimensions measured by the SCL-90 scale than the healthy adults did (27). Elevated depression, anxiety, and interpersonal sensitivity further indicate high vulnerability to emotional distress.

In our study, we also came to a similar conclusion, with somatization (SOM) and psychoticism (PSY) being the two dimensions with the highest scores of the 10 assessed in the group of PWUDs with HCV (Table 2). The high somatization scores suggest that psychological distress frequently manifests as physical complaints, which may be intertwined with the virus's systemic effects (such as fatigue), the physical toll of substance use, or the stress of the diagnosis itself. The elevated psychoticism scores point towards a high prevalence of distorted thought processes and feelings of alienation. This severe distress is likely compounded by factors such as social stigma, the neurological effects of drug use, and the intense pressures of compulsory rehabilitation treatment. Furthermore, the prominent elevations in DEP, ANX, and IS underscore the deep emotional and social challenges faced by this population. These findings are consistent with literature attributing HCV-related depression and anxiety to the social limitations, life stressors, and diminished social support that often follow a diagnosis, particularly within the context of intravenous drug use. Therefore, PWUDs, particularly those with HCV, experience higher rates of severe mental illness compared to the general population.

Ultimately, these results issue a clear call for action. They powerfully argue for the necessity of integrated treatment models that address mental health concurrently with antiviral therapy. Merely treating the virus is insufficient. To improve treatment adherence, reduce substance use relapse, and enhance the overall quality of life, mental health interventions are essential. As other studies have shown, approaches like peer-based support programs and specialized nurse-led services can be highly effective (28). Thus, delivering mental health services may reduce barriers to care and improve outcomes. Studies indicate that peer-based approaches, peer educators, and collaboration among medical providers may facilitate successful HCV screening and treatment among PWUDs. Another paper in Australia indicated that a nurse-led, HCV service for PWUDs with severe mental illness could achieve high treatment uptake and cure (29). In summary, targeted mental health services and peer-based programs can significantly improve treatment outcomes for HCV-positive PWUDs with severe mental illness.

## 5 Limitations

Our study had several limitations that should be emphasized. First, our study did not include individuals who did not identify as PWUDs, limiting our ability to compare HCV-positive PWUDs with HCV patients who do not use drugs. Furthermore, the lack of a general population comparison group limits the external applicability of the high knowledge levels observed. Second, although both the HCV knowledge questionnaire and the SCL-90 demonstrated strong reliability and validity in this population, several caveats remain. The knowledge questionnaire was developed specifically for this study and, despite acceptable KR-20 (0.89) and expert-validated content, further replication in broader PWUD cohorts is warranted. Third, the wording of certain knowledge items, such as the question regarding sexual transmission, may warrant refinement to avoid ambiguity; however, based on standard guidelines, the non-recognition of sexual transmission risk was scored as a knowledge deficit, as it is a confirmed transmission route. Fourth, we did not collect data on the duration since previous diagnosis in HCV-positive patients, which limits our ability to fully interpret the relationship between the length of infection and the level of knowledge among the already aware participants. Finally, participants from the Drug Rehabilitation Center were provided with education about HCV, suggesting that their level of awareness, behavioral tendencies, emotional states, and experiences may not accurately represent PWUDs in the broader community who are not receiving rehabilitation services.

## 6 Conclusion

This study of PWUDs in a Chinese rehabilitation center found that while general HCV knowledge is high, awareness of risks from unregulated cosmetic procedures is poor. Sharing injection equipment remains the primary risk factor. Crucially, HCV-positive PWUDs experience severe psychological distress, highlighting the need for integrated care models that address both virological and mental health needs. To achieve HCV elimination, strategies must expand beyond basic education to include targeted awareness and the incorporation of mental health services for this vulnerable population.

## Data Availability

The raw data supporting the conclusions of this article will be made available by the authors, without undue reservation.
