# Supplementary figures and images for: Assessing the knowledge, attitudes, and psychological health of people who use drugs towards Hepatitis C: a cross-sectional study
Source: Front Public Health. 2025 Oct 31;13:1618440. doi: 10.3389/fpubh.2025.1618440 (PMC12615505; doi:10.3389/fpubh.2025.1618440)

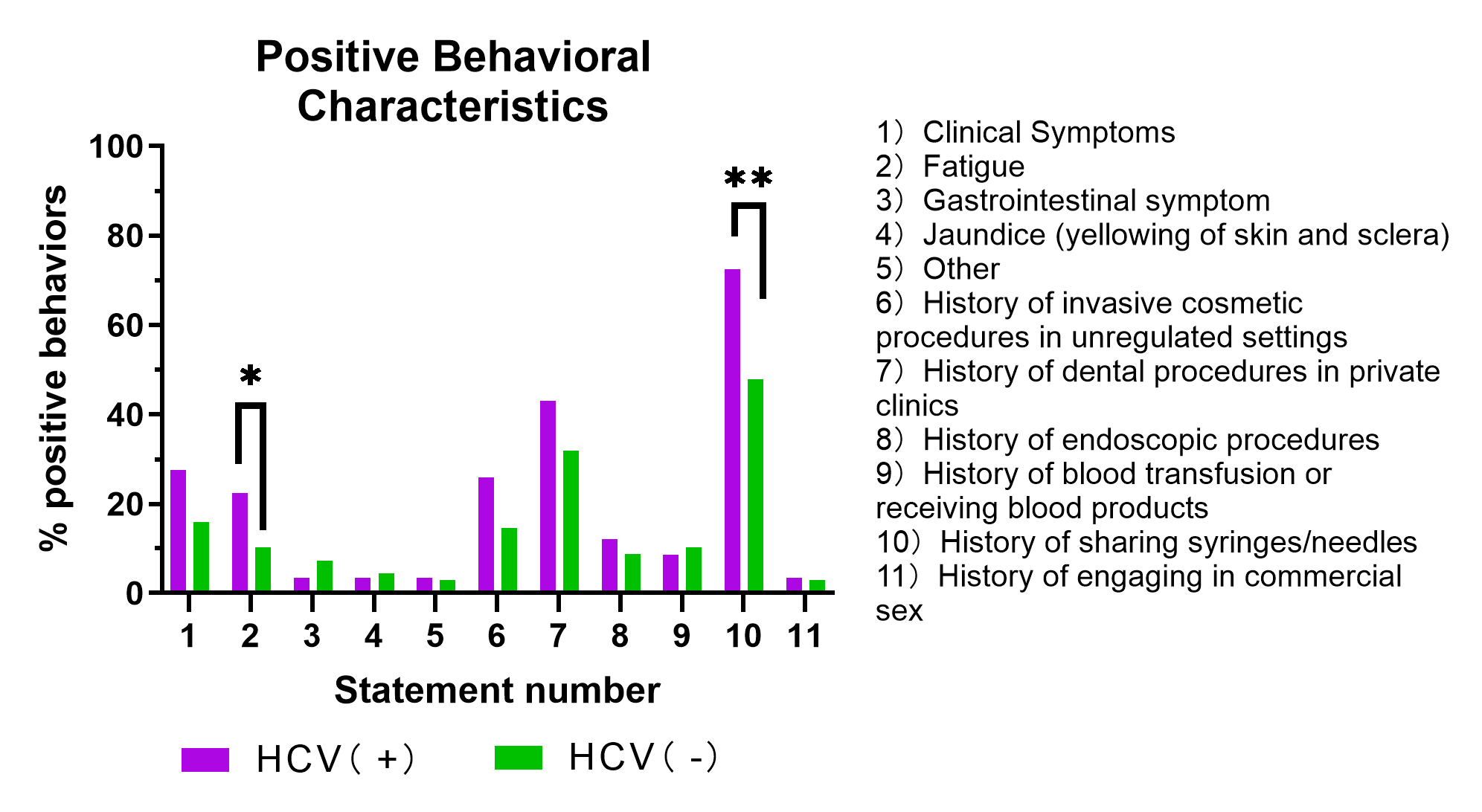

Supplement: Supplementary file 1 [file Data_Sheet_1.zip › HCV attitude.tif]

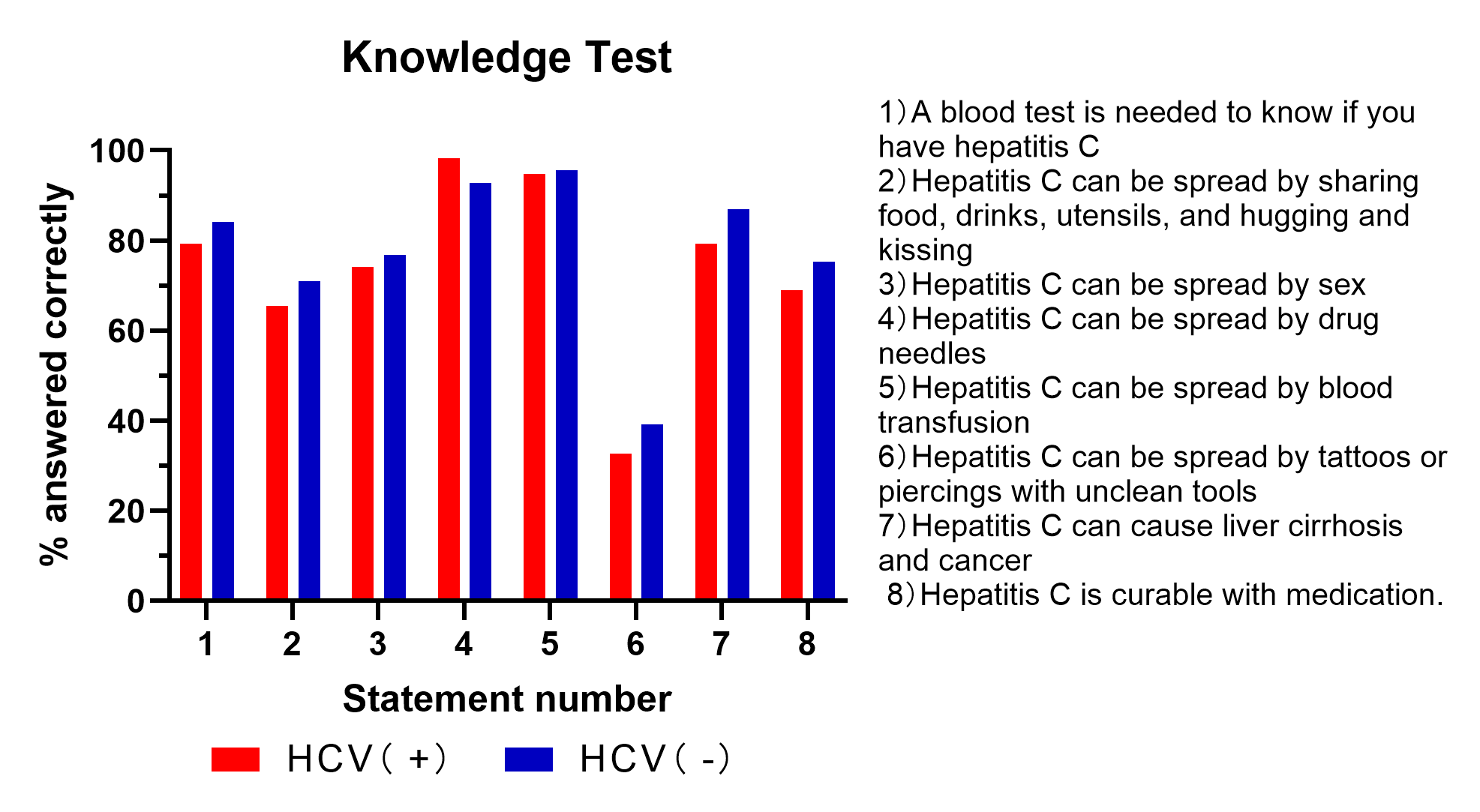

Supplement: Supplementary file 1 [file Data_Sheet_1.zip › hcv knowledge.tif]
